# Prostaglandin metabolite induces inhibition of TRPA1 and channel-dependent nociception

**DOI:** 10.1186/1744-8069-8-75

**Published:** 2012-09-27

**Authors:** Yingqi Weng, Patricia A Batista-Schepman, Marie E Barabas, Eli Q Harris, Thomas B Dinsmore, Elena A Kossyreva, Audra M Foshage, Michelle H Wang, Matthew J Schwab, Victoria M Wang, Cheryl L Stucky, Gina M Story

**Affiliations:** 1 Department of Anesthesiology, Washington University Pain Center, St. Louis, MO 63110, USA; 2 Washington University School of Medicine, 660 S. Euclid Avenue, Campus Box 8054, St. Louis, MO 63110, USA; 3 Department of Anesthesiology, Xiangya Hospital, Central South University, Changsha, Hunan, 43110, China; 4 Department of Pharmacology, Center of Biological Science, Federal University of Santa Catarina, Florianopolis, SC, Brazil; 5 Department of Cell Biology, Neurobiology and Anatomy, Medical College of Wisconsin, Milwaukee, WI, 53226, USA

**Keywords:** TRPA1, 15d-PGJ_2_, Mustard oil, Negative modulation, Mechanical hypersensitivity

## Abstract

**Background:**

The Transient Receptor Potential (TRP) ion channel TRPA1 is a key player in pain pathways. Irritant chemicals activate ion channel TRPA1 via covalent modification of N-terminal cysteines. We and others have shown that 15-Deoxy-Δ12, 14-prostaglandin J_2_ (15d-PGJ_2_) similarly activates TRPA1 and causes channel-dependent nociception. Paradoxically, 15d-PGJ_2_ can also be anti-nociceptive in several pain models. Here we hypothesized that activation and subsequent desensitization of TRPA1 in dorsal root ganglion (DRG) neurons underlies the anti-nociceptive property of 15d-PGJ_2_. To investigate this, we utilized a battery of behavioral assays and intracellular Ca^2+^ imaging in DRG neurons to test if pre-treatment with 15d-PGJ_2_ inhibited TRPA1 to subsequent stimulation.

**Results:**

Intraplantar pre-injection of 15d-PGJ_2_, in contrast to mustard oil (AITC), attenuated acute nocifensive responses to subsequent injections of 15d-PGJ_2_ and AITC, but not capsaicin (CAP). Intraplantar 15d-PGJ_2_—administered after the induction of inflammation—reduced mechanical hypersensitivity in the Complete Freund’s Adjuvant (CFA) model for up to 2 h post-injection. The 15d-PGJ_2_-mediated reduction in mechanical hypersensitivity is dependent on TRPA1, as this effect was absent in TRPA1 knockout mice. Ca^2+^ imaging studies of DRG neurons demonstrated that 15d-PGJ_2_ pre-exposure reduced the magnitude and number of neuronal responses to AITC, but not CAP. AITC responses were not reduced when neurons were pre-exposed to 15d-PGJ_2_ combined with HC-030031 (TRPA1 antagonist), demonstrating that inhibitory effects of 15d-PGJ_2_ depend on TRPA1 activation. Single daily doses of 15d-PGJ_2_, administered during the course of 4 days in the CFA model, effectively reversed mechanical hypersensitivity without apparent tolerance or toxicity.

**Conclusions:**

Taken together, our data support the hypothesis that 15d-PGJ_2_ induces activation followed by persistent inhibition of TRPA1 channels in DRG sensory neurons *in vitro* and *in vivo*. Moreover, we demonstrate novel evidence that 15d-PGJ_2_ is analgesic in mouse models of pain via a TRPA1-dependent mechanism. Collectively, our studies support that TRPA1 agonists may be useful as pain therapeutics.

## Background

Transient receptor potential cation channel subfamily A member 1 (TRPA1) was first identified as a channel activated by noxious cold (~10-17°C) and was thus included in the temperature-gated subfamily termed “thermoTRPs” [1,2]. Whether TRPA1 is directly activated by cold and if it plays a role in thermosensation *in vivo* remain equivocal and debated issues in the somatosensory field [3-5]. However, its activation by a variety of noxious chemicals is widely accepted. The catalogue of TRPA1 chemical agonists is burgeoning and includes a variety of exogenous, as well as endogenous, compounds. We and others identified 15d-PGJ_2_, a multi-functional prostaglandin molecule, as an endogenous TRPA1 activator. Similar to other TRPA1 agonists, intraplantar (ipl.) administration of high concentrations (relative to physiological levels) of 15d-PGJ_2_ causes TRPA1-dependent nocifensive behavior [6-9].

15d-PGJ_2_, one of three J-series prostaglandin D_2_ metabolites, is the most recently discovered prostaglandin with a proposed role as an endogenous anti-inflammatory agent [10]. 15d-PGJ_2_ activates molecules in anti-inflammatory pathways through covalent modification of cysteine residues. This occurs because of its reactive cyclopentenone ring, which readily reacts with nucleophilic cysteine groups through the Michael addition reaction [11]. This has been shown using non-reactive analogues of 15d-PGJ_2_ and by mutagenizing cysteine residues of target proteins such as IkappaB kinase and PPARγ [12-16]. Similarly, cysteine residues of TRPA1 can be modified by certain electrophilic agonists, which leads to activation of the channel by chemicals of this class [11,17,18].

Recent findings support that 15d-PGJ_2_ also exhibits anti-nociceptive properties [9,19,20]. Here we demonstrate a novel TRPA1-dependent anti-nociceptive modality of 15d-PGJ_2_ in acute nociception and mechanical hypersensitivity. Although the pro- and anti-nociceptive effects of 15d-PGJ_2_ may seem mutually exclusive, we propose a mechanism based on our data that reconciles these seemingly opposing effects. We hypothesize that 15d-PGJ_2_ is anti-nociceptive owing in part to its ability to activate and desensitize TRPA1 in peripheral nociceptive fibers.

Our findings support this hypothesis. Peripheral injection of a pro-nociceptive and behaviorally desensitizing dose of 15d-PGJ_2_ produces an attenuation of acute nocifensive behavior induced by AITC, whereas AITC itself does not produce such effects. Correspondingly, we find that 15d-PGJ_2_ produces a marked inhibition of subsequent responses to AITC in DRG neurons. When administered after the induction of inflammation, 15d-PGJ_2_ reduces mechanical hypersensitivity in WT but not TRPA1 knockout (TRPA1^−/−^) mice, arguing that these analgesic effects are mediated via the channel. Taken together our data suggest that 15d-PGJ_2_ induces a reduction of chemical and mechanical nociception via initial activation and subsequent inhibition of TRPA1. Results also indicate that this property may be unique to 15d-PGJ_2_ as an endogenous TRPA1 activator, as AITC did not have the same effects in behavioral assays or in DRG neurons.

## Results

### Effects of 15d-PGJ_2_ on mechanosensitivity

We hypothesized that 15d-PGJ_2_ is anti-nociceptive owing in part to its ability to activate, and subsequently desensitize, TRPA1. We set out to test whether 15d-PGJ_2_ is anti-nociceptive in pain models in which a role of TRPA1 is implicated. After the induction of mechanical hypersensitivity by CFA, TRPA1-selective antagonists AP-18 and HC-030031 ameliorate post-CFA mechanical thresholds in WT but not TRPA1^−/−^ mice [21-23]. Therefore, we utilized the CFA model to investigate whether 15d-PGJ_2_ could reverse inflammatory mechanical hypersensitivity.

**Figure 1:**
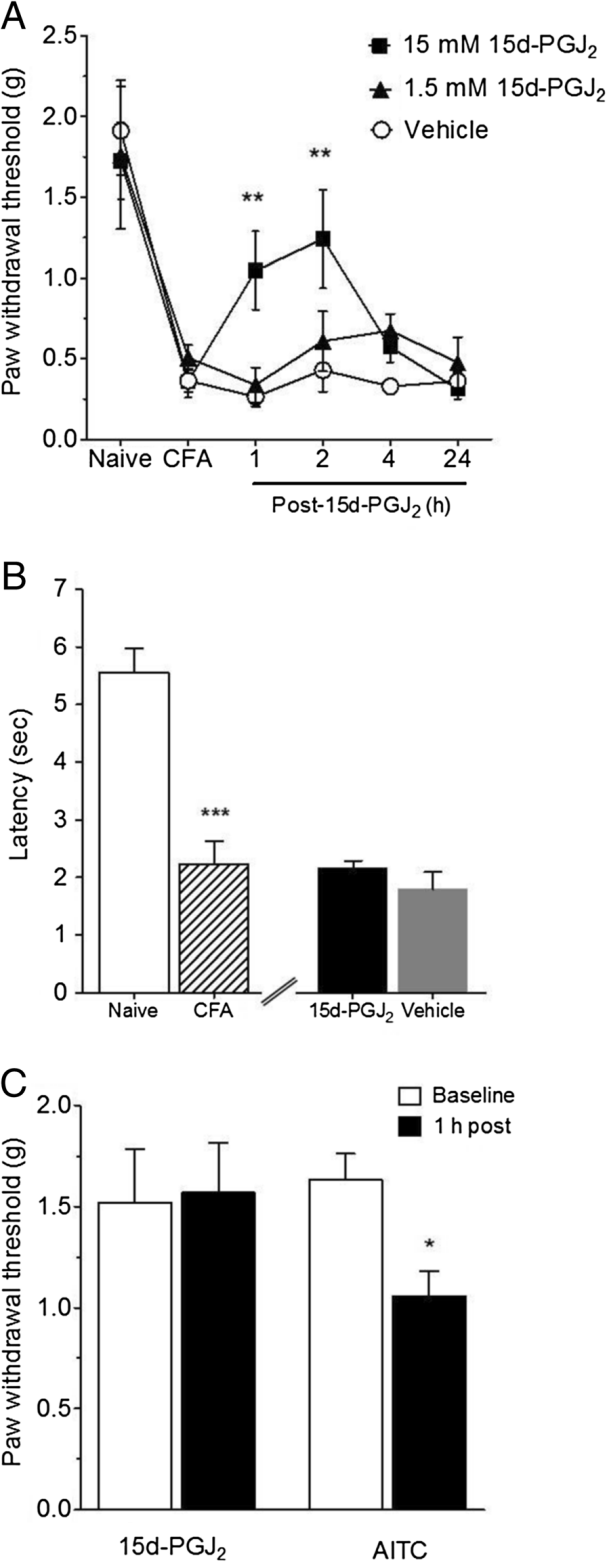
**Effect of 15d-PGJ**_**2 **_**on mechanical- and heat-sensitivity. **(**A**) 15 mM 15d-PGJ_2 _(ipl.) attenuates CFA-induced mechanical hypersensitivity. Von Frey measurements were recorded before CFA injection (Naïve), 24 h post-CFA injection and at noted time points. 15d-PGJ_2_ was administered 1 h prior to von Frey measurements. (**B**) 15d-PGJ_2 _shows no analgesic effect (no change latency to licking or flicking) against inflammatory heat hypersensitivity in Hargreaves test. (**C**) 15d-PGJ_2 _itself does not cause mechanical hypersensitivity (1 h after ipl. injection), while AITC does (*p < 0.05, **p < 0.01, *** *p* < 0.001; n = 8 per group; values expressed as mean ± SEM). Data were analyzed using RMANOVA with Bonferonni post-hoc comparisons (**A**) or two-tailed Student’s t-test (**B **and **C**).

In separate groups of mice, we measured mechanical thresholds using the up and down method at baseline and 24 h post-CFA injection (day 1, Figure 1A). One day after CFA injection, we injected 1.5 or 15 mM 15d-PGJ_2_ (10 μL) into the plantar hindpaw 1 h prior to von Frey measurements. As shown in Figure 1A, 15 mM 15d-PGJ_2_ induced a marked reversal of mechanical hypersensitivity relative to vehicle (and 1.5 mM 15d-PGJ_2_). The effect was maximal at 2 h post injection. In contrast, 15d-PGJ_2_ had no effect on heat hypersensitivity as measured with the Hargreaves assay, suggesting that the effect of 15d-PGJ_2_ is modality specific (Figure 1B). Administering 15d-PGJ_2_ prior to the induction of inflammation by CFA had no effect on mechanical thresholds in CFA- or vehicle-injected mice (data not shown, see also below).

Previous studies characterizing the analgesic effects of 15d-PGJ_2_ do not report whether the compound alters baseline mechanical sensitivities in naïve/un-injured rodents [9,19,20]. Therefore, we also investigated whether ipl. injection of 15 mM 15d-PGJ_2_, administered 1 h prior to von Frey testing, caused any change in mechanical thresholds relative to vehicle-injected controls. As shown in Figure 1C, mechanical sensitivities of 15d-PGJ_2_ and vehicle-injected mice did not significantly differ. This indicates that 15d-PGJ_2_ does not generate mechanical hypo- or hypersensitivity. In contrast, pre-injection of an equivalent concentration of AITC (15 mM) caused a significant reduction in paw withdrawal threshold, indicative of hyperalgesia (Figure 1C). These results are similar to studies showing that AITC and cinnamaldehyde induce significant mechanical hyperalgesia in rats [24,25]. Furthermore, our data also suggest disparate *in vivo* effects of the two TRPA1 agonists 15d-PGJ_2_ and AITC.

### 15d-PGJ_2_ and AITC evoke equivalent nocifensive behavior

**Figure 2:**
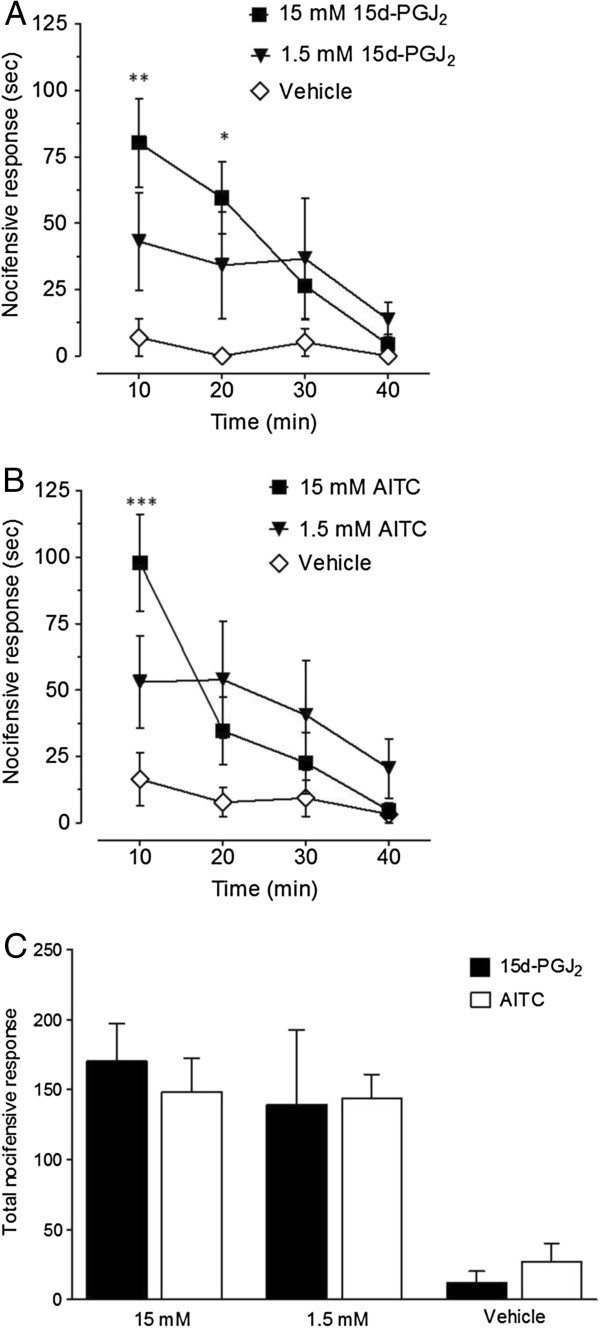
**Dose–response of nocifensive behavior evoked by 15d-PGJ**_**2 **_**or AITC. **Mice were administered (10 μL ipl.) 15d-PGJ_2 _(**A**) or AITC (**B**). Licking and deliberate lifting of the injected hindpaw was recorded at 10-min intervals for 40 min. (**C**) Total nocifensive response for 15d-PGJ_2 _vs. AITC are equivalent at 1.5 and 15 mM. (*p < 0.05, **p < 0.01 for 15 mM 15d-PGJ_2 _vs. vehicle at 10 and 20 min time points, respectively; ***p < 0.01 for 15 mM ATIC vs. vehicle; n = 6-8 per group; values expressed as mean ± SEM). Data were analyzed using RMANOVA with Bonferonni post-hoc comparisons.

We reasoned that the different pharmacological effects of 15d-PGJ_2_ and AITC could be due to their relative potencies *in vivo*. Though the two TRPA1-specific compounds have similar *in vitro* EC_50_ values, *in vivo* potencies have not been directly compared [7]. Therefore, we performed dose–response analysis using two concentrations of 1.5 and 15 mM 15d-PGJ_2_ or AITC (10 μL, ipl.). To determine both the magnitude and duration of nocifensive behaviors at each dose of the two compounds, we recorded at 10-min intervals for 1 h. Figure 2 illustrates the total nocifensive response at 10-min increments (A-B) and the total duration (C) of nocifensive responses during the course of 40 min at each concentration of 15d-PGJ_2_ and AITC. 15d-PGJ_2_- and AITC-induced nocifensive behaviors consisted of licking, deliberate lifting and shaking of the injected hindpaw. Nocifensive behaviors subsided approximately 40 min after injection of 15 mM or 1.5 mM of 15d-PGJ_2_ or AITC (Figure 2 A-B, data not shown). The efficacy of 15 and 1.5 mM 15d-PGJ_2_ and AITC in producing total nocifensive responses were equivalent (Figure 1C). For both 15d-PGJ_2_ and AITC, 0.15 mM doses did not induce significant nocifensive behaviors compared to vehicle (data not shown). The 0.15 mM dose of 15d-PGJ_2_ (475 ng/paw) is similar to another study (300 ng/paw), which also did not report nocifensive behavior [19].

### Behavioral desensitization of 15d-PGJ_2_ versus AITC

Previously it was reported that the second response to ipl. injection of 0.1% (10 mM) AITC, administered 15 min after the first injection, is significantly reduced in rats [26]. However, another more recent study in mice demonstrated that nocifensive responses to the second injection of 10 mM AITC, administered 10 min after the first, sensitize significantly [27]. The discrepancy between these two studies could arise from differences in dosing protocols or due to species differences. We determined whether 15d-PGJ_2_ inhibits TRPA1-dependent nociception *in vivo* via a simple cross-desensitization mechanism and whether this property of 15d-PGJ_2_ is distinct from AITC. Our experiment was designed to more carefully examine behavioral desensitization of 15d-PGJ_2_ vs. AITC responses *in vivo*. We used a 1 h inter-stimulus interval to correspond with our dosing paradigm(s) in the above CFA model. In addition, our design aimed to ensure that nocifensive responses to the first dose of both compounds had completely subsided prior to administering the second dose.

**Figure 3:**
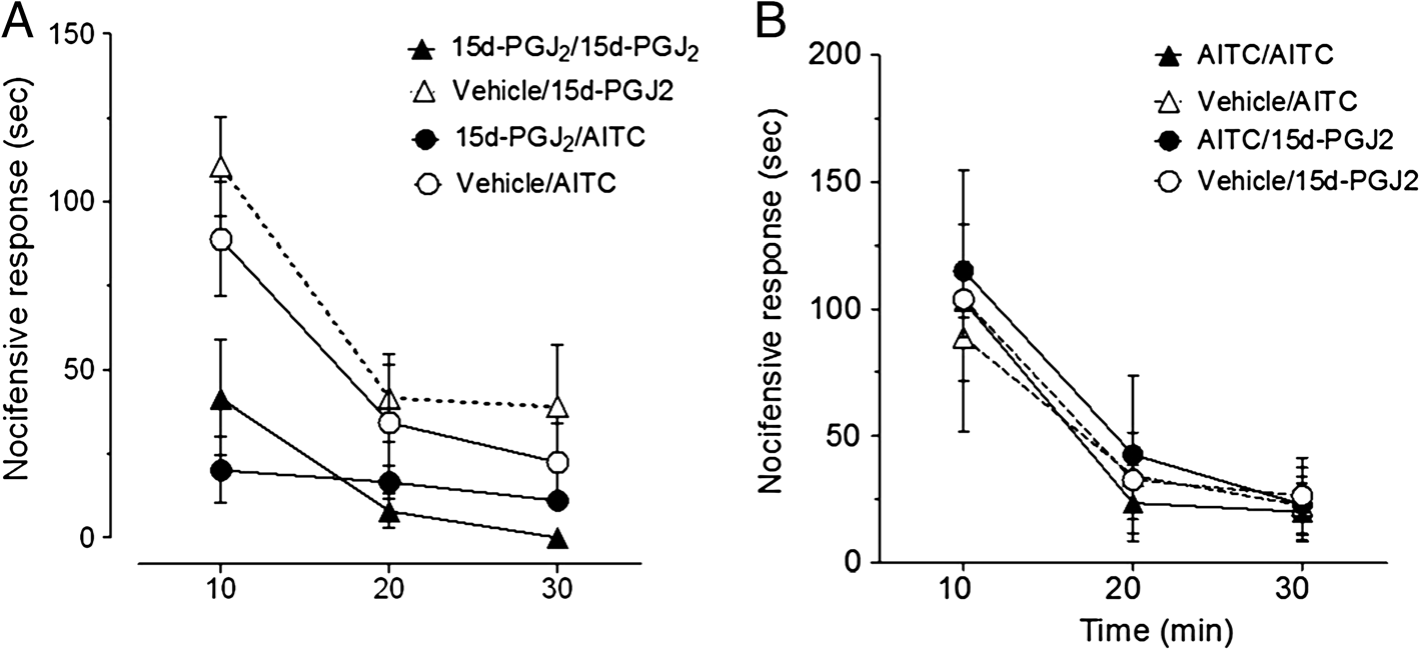
**Homologous and heterologous desensitization of 15d-PGJ**_**2 **_**vs. AITC. **Multi-dose experiments 15d-PGJ2 (**A**) or AITC (**B**) cause homologous or heterologous desensitization in vivo, we performed multi-dose experiments. The inter-stimulus interval was 1 h. Pre-injection (10 μL ipl.) of vehicle was used as control. Behavior was recorded at 10-min intervals for 30–40 min. (**A**) Nocifensive response to 15 mM 15d-PGJ_2 _or 50 mM AITC after 15 mM 15d-PGJ_2 _pre-injection was significantly reduced relative to vehicle pre-injected. (Not shown for Figure clarity: *p < 0.05 for 15 mM 15d-PGJ_2_/15d-PGJ_2 _vs. 15d-PGJ_2_/vehicle at 10 and 20 min time points; **p < 0.01 for 15d-PGJ_2_/AITC vs. vehicle/AITC at 10 min time point (**B**) Nocifensive responses to the injection of either 15 mM AITC or 15d-PGJ_2 _were not reduced by 15 mM AITC pre-injection relative to vehicle controls. (n = 8 animals per group; Values expressed as mean ± SEM). Data were analyzed using RMANOVA with Bonferonni post-hoc comparisons.

We first examined desensitization of behavioral responses to repeated 15d-PGJ_2_ stimuli and tested whether 15d-PGJ_2_ could inhibit subsequent responses to AITC. Unlike the AITC multi-dose results (Figure 3B), two injections (10 μL each, ipl.) of 15 mM 15d-PGJ_2_ produced significant reductions of the second nocifensive response to 15 mM 15d-PGJ_2_ and to AITC at 15 mM and at concentrations as high as 50 mM (Figure 3A and data not shown). The anti-nociceptive effect of 15 mM 15d-PGJ_2_ was locally mediated as ipl. injection in the contralateral hindpaw 1 h prior to ipl. injection of AITC did not significantly reduce AITC-induced nocifensive behavior (total nocifensive response 83 ± 15 sec vs. 97 ± 7 sec, *p* > 0.05).

Next, we tested whether 15 mM AITC could inhibit nocifensive responses to a subsequent injection of 15 mM 15d-PGJ_2_ via cross-desensitization. Pre-injection of 15 mM AITC did not induce cross-desensitization of behavioral responses to 15 mM 15d-PGJ_2_ (10 μL each, ipl.) (Figure 3B). We also did not observe homologous behavioral sensitization or desensitization as the nocifensive response to a second injection of 15 mM AITC was equivalent to that evoked by 15 mM AITC administered 1 h prior (and relative to vehicle pre-injection) (Figure 3B). The highest tested dose of AITC (50 mM) also did not inhibit nocifensive responses to a subsequent injection of 15 mM 15d-PGJ_2_ (1 h inter-stimulus; data not shown). These data demonstrate that behavioral desensitization to a second TRPA1-dependent noxious stimulus is not due to the noxious nature of the first stimulus. These data suggest that 15d-PGJ_2_ induces behavioral desensitization to itself and to AITC. In contrast, ATIC does not induce desensitization to itself or 15d-PGJ2.

### 15d-PGJ_2_ vs. AITC inhibition in DRG

In our previous publication characterizing 15d-PGJ_2_ activation of TRPA1-expressing DRG neurons, we showed that 15d-PGJ_2_ activated ~19% of total DRG neurons and that 98% of these also responded to AITC. We also demonstrated that 15d-PGJ_2_ responses by DRG neurons were absent in cultures derived from TRPA1^−/−^ mice [6]. Collectively, these data indicate that 15d-PGJ_2_ is a selective agonist at TRPA1. We further hypothesized that a potential mechanism for the reported anti-nociceptive properties of 15d-PGJ_2_[9,19,20] is excitation and subsequent inhibition/desensitization of DRG neurons expressing TRPA1. Therefore, in order to evaluate whether 15d-PGJ_2_ could desensitize TRPA1-expressing nociceptors to other agonists, we utilized Fura-2 Ca^2+^ imaging in DRG neurons, to compare responses to 15d-PGJ_2_ and AITC in vehicle-, 15d-PGJ_2_- and AITC- pre-stimulated cultures.

**Figure 4:**
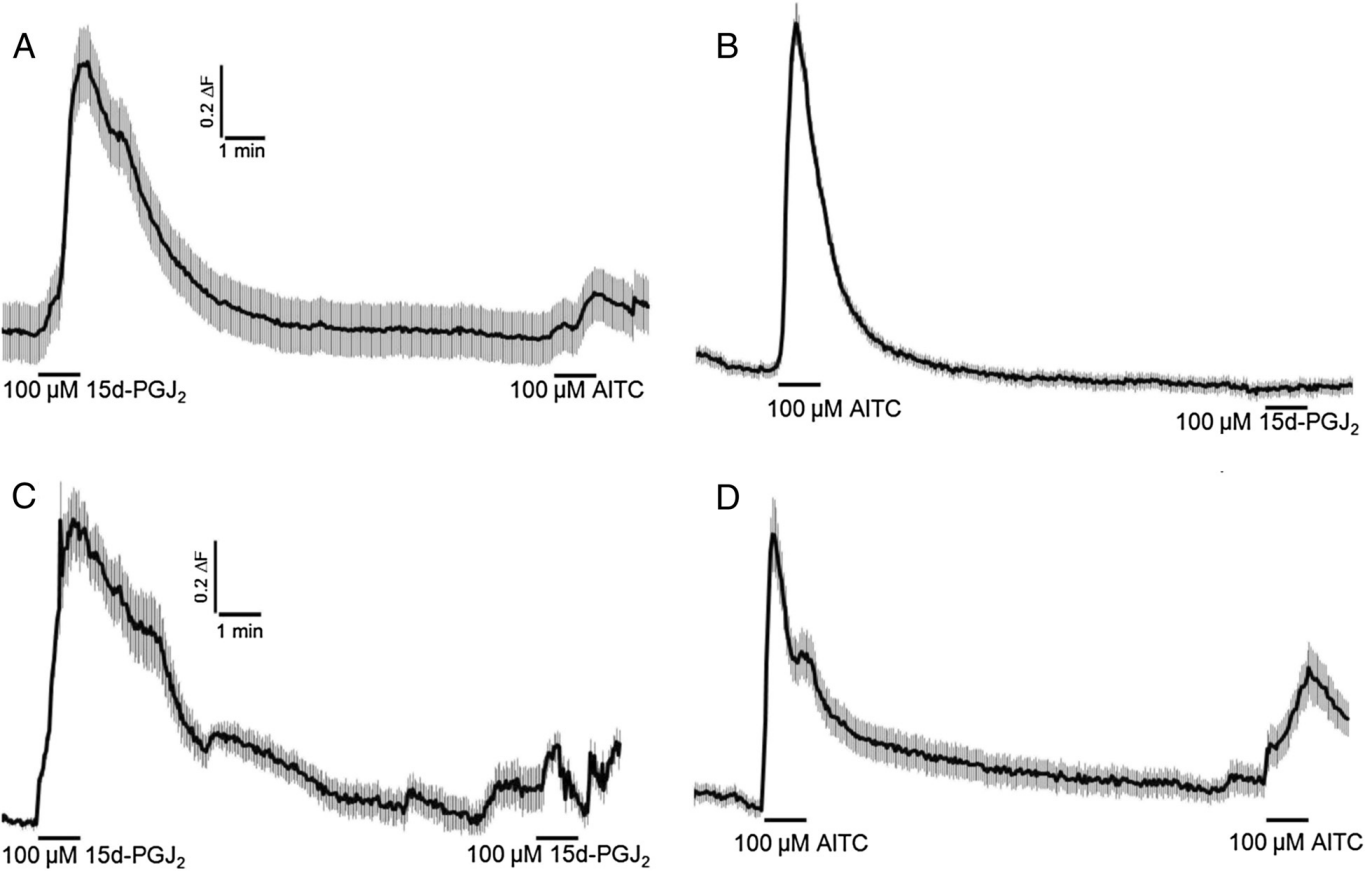
**Homologous and heterologous inhibition evoked by 100 μM 15d-PGJ**_**2 **_**vs. 100 μM AITC. **A pre-pulse of 100 μM 15d-PGJ_2_ desensitized subsequent responses to 100 μM AITC (**A**) and (**B**). Similarly, 100 μM AITC desensitized subsequent responses to 100 μM 15d-PGJ_2 _and 100 μM AITC. Interstimulus intervals of 4–8 minutes were tested (8 min shown in **A**-**D**). Traces show mean ± SEM of 10–30 neurons.

**Figure 5:**
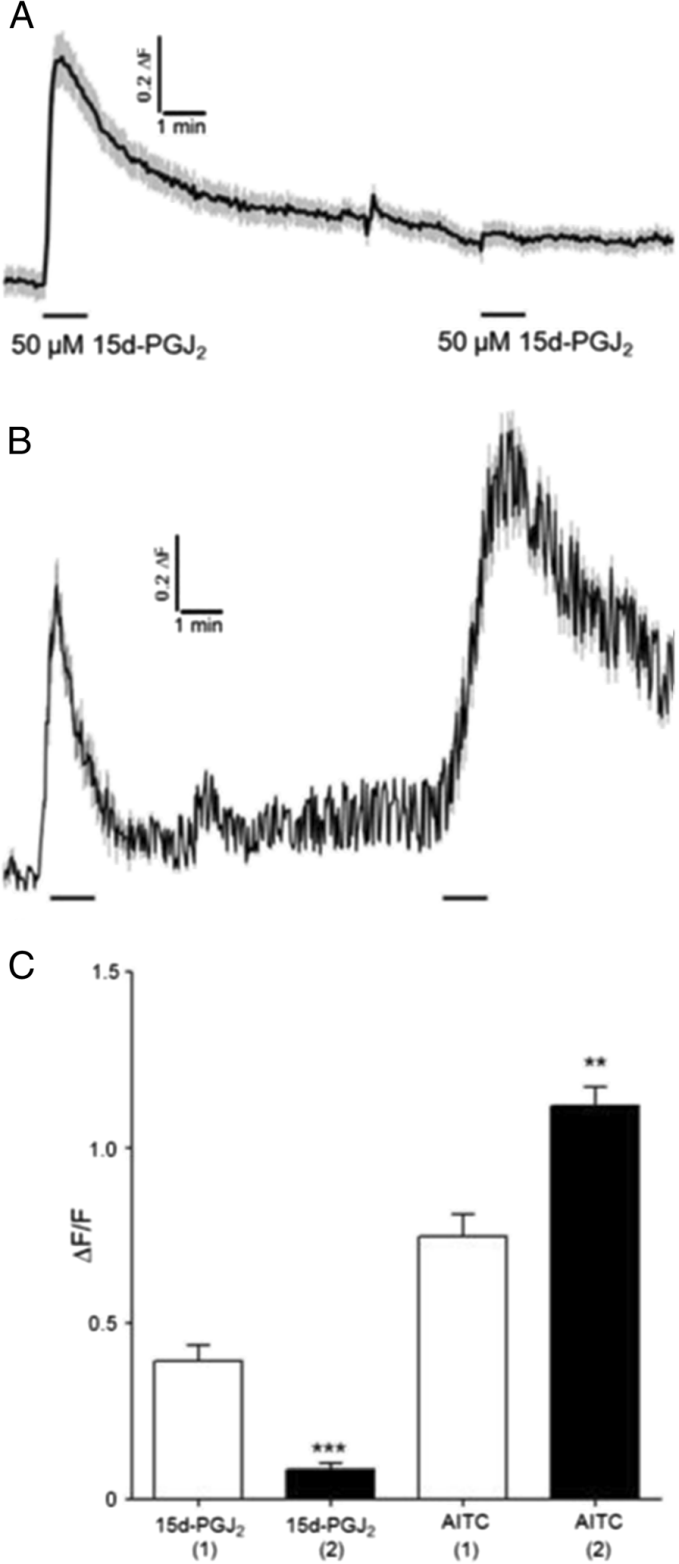
**Homologous desensitization of 15d-PGJ**_**2 **_**vs. AITC is dependent on agonist concentration. **Homologous sensitization and desensitization are agonist- and concentration-dependent. In contrast to 50 μM 15d-PGJ_2 _(**A**), responses to 50 μM AITC sensitize (**B**) significantly (**C**). Traces show mean ± SEM of ~30-60 DRG neurons. Values in C represent the peak response of neurons under each condition. **p < 0.01, ***p < 0.001. Data were analyzed using a paired, two-tailed Student’s t-test.

We conducted cross- and homologous-inhibition experiments utilizing two bath applied pulses of 50–100 μM 15d-PGJ_2_ or AITC. As shown in Figure 4, 100 μM 15d-PGJ_2_ strongly inhibits subsequent responses to a pulse of 100 μM AITC applied up to 8 min later (Figure 4A) and to itself (Figure 4B). Similarly, 100 μM ATIC strongly desensitizes subsequent responses to 100 μM 15d-PGJ_2_ (Figure C) and to itself (Figure 4D). Cross-desensitization was maintained when we applied two pulses of 50 μM of each compound (data not shown). In experiments utilizing two 50 μM pulses of 15d-PGJ_2_ or two 50 μM pulses of AITC, homologous desensitization of 15d-PGJ_2_, but not AITC, was maintained (Figure 5). With up to an 8 min inter-stimulus interval, two 50 μM 15d-PGJ_2_ pulses revealed a significant homologous desensitization of the second 15d-PGJ_2_ response (Figure 5A). Intriguingly, the reverse was not evident—50 μM AITC did not inhibit the percentage of neurons responding (39.7% vs. 38.2%) or reduce the overall magnitude of responses to a subsequent stimulus of 50 μM ATIC (Figure 5B). Instead, the second response to 50 μM AITC was significantly sensitized relative to the first (Figure 5C). These experiments reveal complex concentration- and stimulus-dependent variables that affect TRPA1 activation.

### 15d-PGJ_2_ inhibits AITC, but not CAP responses

**Figure 6:**
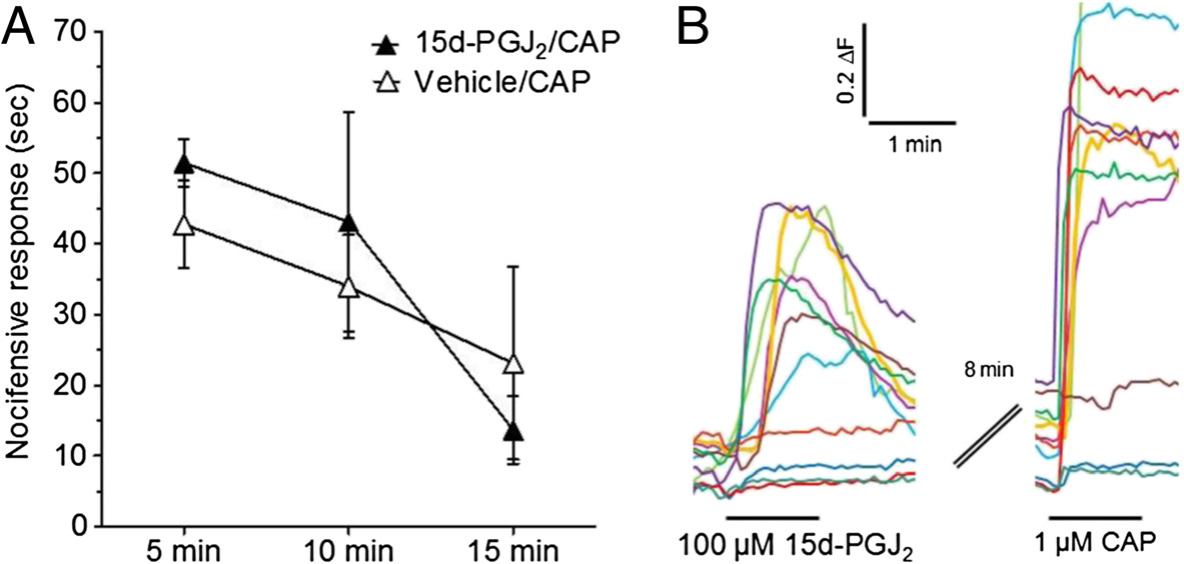
**15d-PGJ**_**2 **_**does not affect cellular or behavioral responses to CAP. **(**A**) 100 μM 15d-PGJ_2 _did not affect the response magnitude induced by 1 μM CAP (**B**). In agreement with calcium imaging of DRG data, pre-treatment with 15 mM 15d-PGJ_2_ (10 μL ipl.) did not inhibit behavioral responses to an 1.6 μg CAP (10 μL ipl.).

Previous behavioral studies in rats demonstrated heterologous desensitization between TRPA1 and TRPV1 *in vivo*[26,28]. A pre-injection of 10 mM (0.1%) AITC administered 15 min prior to 10 μg CAP, significantly reduces CAP-induced nocifensive behavior compared to vehicle pre-treatment [26]. To investigate whether 15d-PGJ_2_ could desensitize CAP responses *in vivo*, we utilized the same injection protocol as with our 15d-PGJ_2_ and AITC desensitization studies, injecting 15 mM 15d-PGJ_2_ 1 h prior to 1.5 μg CAP. We did not observe 15d-PGJ_2_-induced behavioral heterologous desensitization of CAP *in vivo* (Figure 6A).

Next, we investigated whether 15d-PGJ_2_ can directly inhibit CAP-responsiveness of DRG neurons. TRPA1 is expressed in a subset of TRPV1-expressing neurons [1]. Several studies have documented the phenomenon of heterologous desensitization between TRPA1 and TRPV1 using heterologous expression systems and cultured sensory neurons [26,28-30]. To determine whether 15d-PGJ_2_ can inhibit subsequent responses to CAP, we pre-exposed DRG neurons to 100 μM 15d-PGJ_2_ vs. vehicle and compared the number and magnitude of CAP responders in the two groups. As illustrated in Figure 6B, 15d-PGJ_2_ did not inhibit CAP responses relative to 0.1% DMSO (vehicle). Neither the total number of neurons responding to CAP (66% vs. 58% of total neurons, *p* > 0.5) nor the magnitude of these responses (ΔF/F = 4.2 vs. 3.8, *p* > 0.5) was affected by pre-application of 15d-PGJ2 relative to vehicle. These findings correlate with our behavioral results and indicate that 15d-PGJ_2_ does not produce heterologous inhibition of TRPV1 via its activation of TRPA1.

### Effects of 15d-PGJ_2_ are TRPA1-dependent

Previous studies have applied pharmacological inhibition and gene knockout approaches to characterize the role of TRPA1 in mechanohypersensitivity *in vivo*. Studies using TRPA1^−/−^ mice suggest that TRPA1 is not involved in the induction of nerve injury- or inflammation-induced hypersensitivity, as TRPA1^−/−^ mice develop hypersensitivity equivalent to WT. However, TRPA1 antagonists (AP-18 and HC-030031) ameliorate post-insult mechanical thresholds in WT but not TRPA1 ^−/−^ mice [21,22].

**Figure 7:**
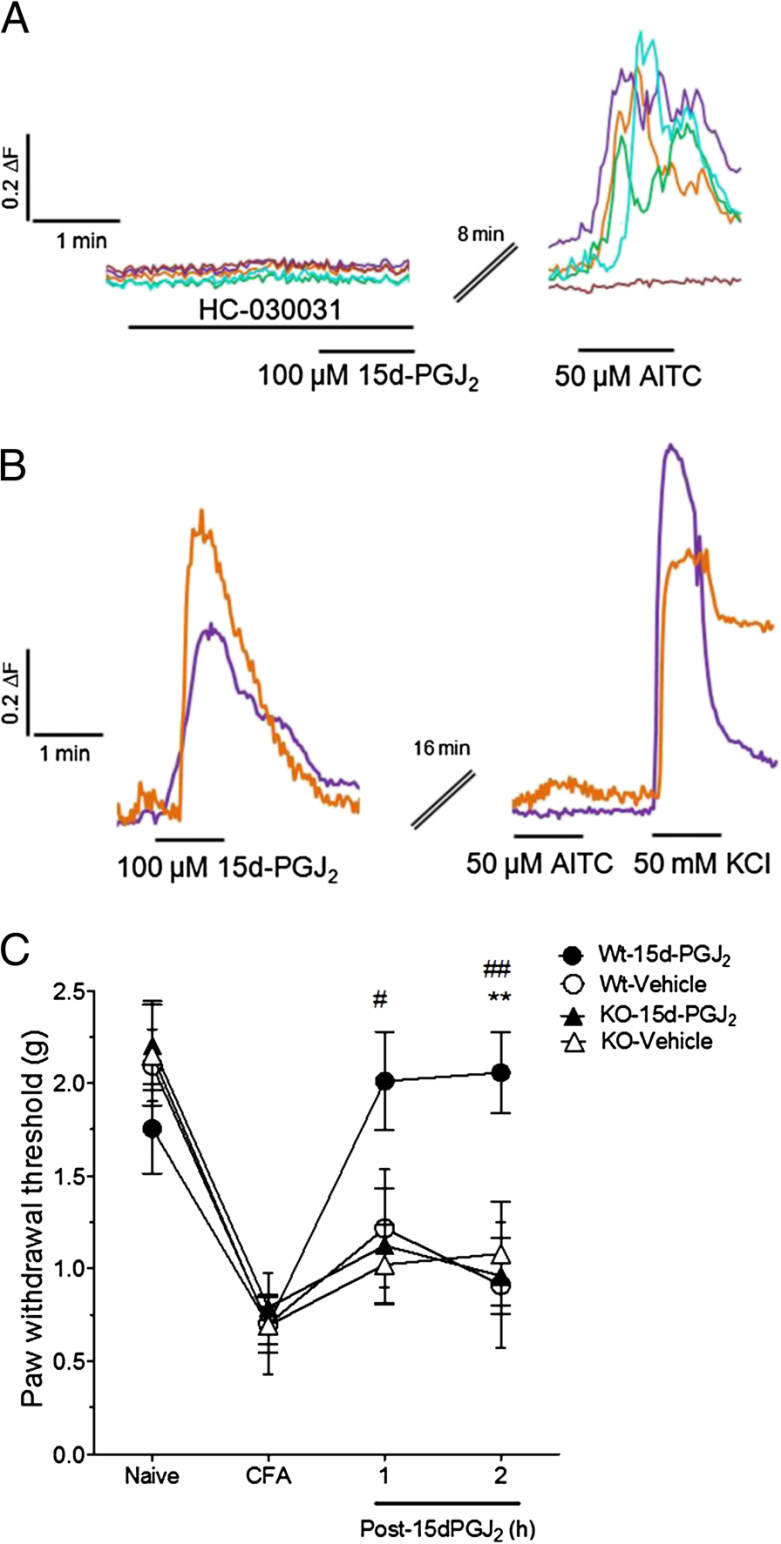
**TRPA1-dependent 15d-PGJ**_**2**_**-mediated inhibition of AITC *in vitro *and mechanical hypersensivity *in vivo*. **(**A**) In the presence of a specific TRPA1 antagonist, HC-030031 (100 μM), 100 μM 15d-PGJ_2 _did not block 50 μM AITC responses. (**B**) We also examined the reversibility of AITC-response inhibition in DRG neurons by 15d-PGJ_2_. 100 μM 15d-PGJ_2 _inhibited subsequent responses to 50 μM AITC for up to 16 min with continuous washout. Traces depict representative cells from 1 or more experiments in which >100 neurons were analyzed per experimental condition. (**C**) 15d-PGJ_2 _(10 μL ipl.) attenuates CFA-induced mechanical hypersensitivity in WT but not TRPA1 −/− mice. Vehicle or 15 mM 15d-PGJ_2 _was injected into the hindpaw of TRPA1 or WT littermates (n = 8 per group) 1 h prior to von Frey measurements. **p < 0.01 in comparing WT mice injected with 15d-PGJ_2 _vs. WT mice injected with vehicle. ^#^*p* < 0.05, ^##^*p* < 0.01 in comparing WT mice injected with 15d-PGJ_2 _vs. TRPA1 KO mice injected with 15d-PGJ_2_. Values expressed as mean ± SEM. Data were analyzed using RMANOVA with Bonferonni post-hoc comparisons.

To address the possibility that 15d-PGJ_2_ inhibits DRG neuron responses to AITC via a mechanism separate from TRPA1 activation, we performed co-application studies in DRG using HC-030031, a selective TRPA1 antagonist. After a 4 min exposure to 100 μM 15d-PGJ_2_ in the presence of antagonist, 50 μM AITC responses of DRG neurons were equivalent to those after vehicle exposure (Figure 7A and data not shown). These data support that the inhibitory effect of 15d-PGJ2 pre-application on DRG neuron responses to AITC is mediated via TRPA1. We also examined the reversibility of desensitization of TRPA1 in DRG neurons by 15d-PGJ_2_. As shown in Figure 7B, 100 μM 15d-PGJ2 inhibited subsequent responses to 50 μM AITC for up to 16 min (4–16 min intervals tested) with continuous washout, while 50 mM KCl responses were unaltered, indicating that general neuronal viability remained intact.

Next we determined whether the observed reduction in CFA-induced mechanical hypersensitivity was due to a TRPA1-specific effect of 15d-PGJ_2_ by repeating the CFA model in groups of TRPA1^−/−^ and WT mice. Whereas 15d-PGJ_2_ caused a rebound of mechanical sensitivities toward baseline in WT mice, this effect was completely absent in TRPA1^−/−^ mice (Figure 7C). These data suggest that the reduction of inflammatory hypersensitivity by 15d-PGJ_2_ is dependent on TRPA1 and taken together our data demonstrate a mechanism involving channel desensitization.

### 15d-PGJ_2_ tolerance and neurotoxicity

**Figure 8:**
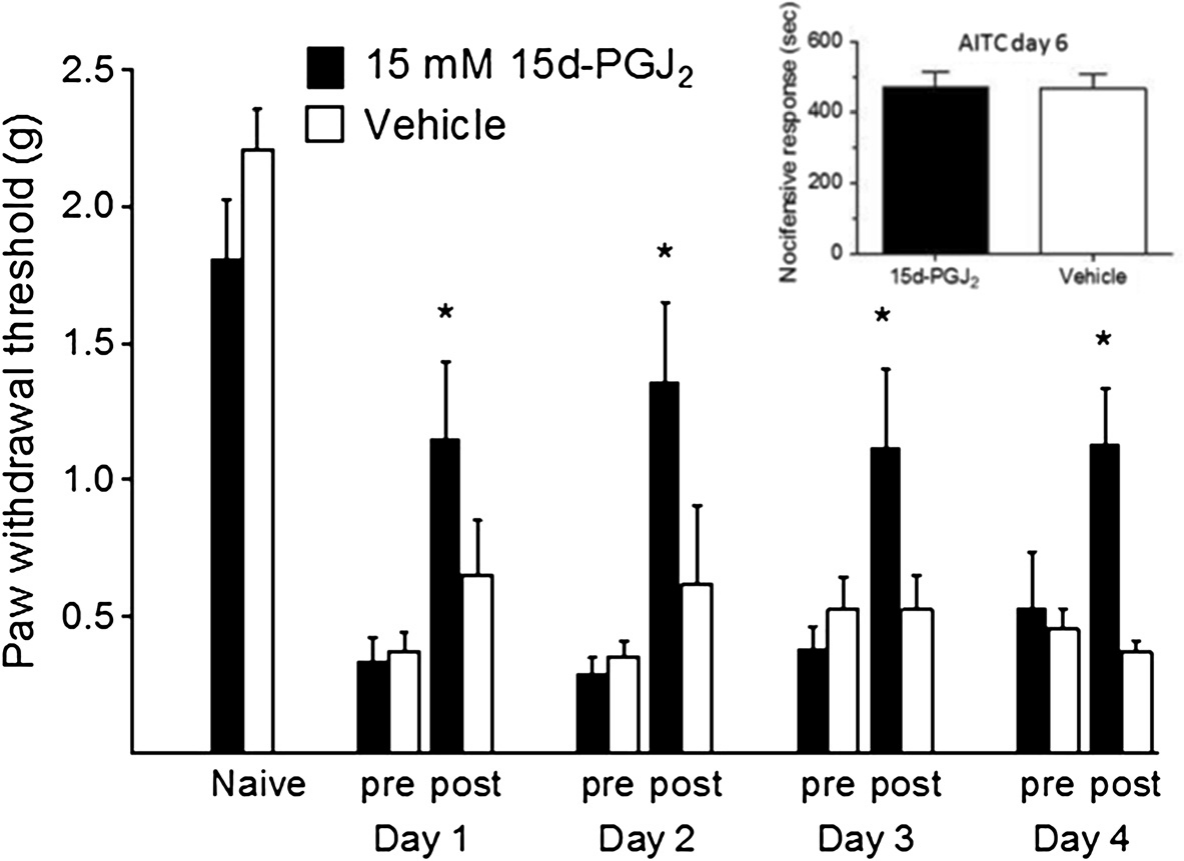
**Multi-dose therapy using 15d-PGJ**_**2 **_**without tolerance or toxicity. **Repeated ipl. injection (1 per day) of 15 mM 15d-PGJ_2 _attenuates CFA- induced mechanical hypersensitivity. Note a significant rebound of mechanical thresholds toward baseline level on days 1–4 (*p < 0.05, n = 6 per group, values expressed as mean ± SEM). At Day 6 AITC responses are intact (inset). Data were analyzed using paired, two-tailed Student’s t-tests.

In order to evaluate the therapeutic utility of 15d-PGJ_2_ in long-term inflammatory hypersensitivity, we utilized a multi-dose protocol. We tested whether repeated doses of 15d-PGJ_2_, administered over the course of days, would continue to be effective in reducing CFA-induced inflammatory mechanical hypersensitivity. As shown in Figure 8, single daily doses of 15 mM 15d-PGJ2 (10 μL, ipl.) repeatedly attenuated mechanical hypersensitivity. To rule out that this effect was due to toxicity or damage of TRPA1-expressing peripheral fibers, we compared AITC responses 48 h after the last dose of 15d-PGJ_2_. We detected no difference in nocifensive behaviors of vehicle- vs. 15d-PGJ_2_-injected groups in response to an injection of 50 mM AITC (10 μL, ipl.) (Figure 8 inset; also note potentiation of AITC response in CFA model).

## Discussion

The primary hypothesis driving our investigation described here is that pre-treatment with 15d-PGJ_2_ protects against TRPA1-mediated nociception and hypersensitivity via inhibition of TRPA1 signaling in nociceptive neurons. Our behavioral data correspond to our observations in DRG sensory neurons. Our results obtained using the acute AITC model suggest that 15d-PGJ_2_ is sufficient to inhibit acute TRPA1-dependent nociception. Moreover, our findings support that a TRPA1-dependent mechanism contributes to the ability of 15d-PGJ_2_ to reduce established inflammatory mechanical hypersensitivity. Finally, we have probed the utility of 15d-PGJ_2_ (a TRPA1 agonist) as a potential therapeutic and find that analgesic effects are maintained over the course of days with repeated dosing.

### Physiological relevance

All TRPA1 agonists identified to date induce nociceptive behavior in rodents or burning sensations in humans (for review) [31-33]. Similarly, we and others have shown that 15d-PGJ_2_ (10–47.4 μg/paw) causes robust nocifensive behavior in WT, but not TRPA1^−/−^, mice [6,7]. These data suggest that 15d-PGJ_2_ (at these concentrations) induces nocifensive behavior that is specific to TRPA1 activation. In contrast, several studies have identified potent anti-nociceptive properties of 15d-PGJ_2_. These studies do not report nocifensive behavior in response to intrathecal (100 μg/site), intraganglionar (100 ng/site), ipl. (30–300 ng/paw) or temporomandibular joint (10–100 ng/site) injection of 15d-PGJ_2_. Concentrations of 15d-PGJ_2_ shown to inhibit nociception in these studies are typically lower than those we have tested here (474 ng-47.4 μg/paw) [6,9,19,20].

In our view, it remains unclear whether the high concentrations of endogenous ligands we and others have tested for their nociceptive- and anti-nociceptive effects are physiologically relevant to pain modulation or to TRPA1 modulation *in vivo*. For example, inflammatory exudate concentrations of 15d-PGJ_2_ rise coincident to the resolution of the inflammatory state. Levels detected range between ~0.5 to 5 ng/ml—orders of magnitude lower than those we and others have potentially introduced by ipl. injection [34,35]. There is currently no *in vitro* data on the time course or kinetics of occupancy of TRPA1 receptors by 15d-PGJ_2_. We are actively attempting to investigate this question and resolve the putative differences in agonist modulation at the channel level. We also currently have no method of ascertaining whether we are actively inhibiting TRPA1 channels at sub-cutaneous nociceptive fibers at the concentration(s) and dosing paradigm(s) we have utilized. However, 15d-PGJ_2_ is not broken down further enzymatically or non-enzymatically. Though it is a reactive electrophile, 15d-PGJ_2_ is highly stable in aqueous phase; when aqueous solutions are spiked with radioactively labeled 15d-PGJ_2_, the compound is 100% recoverable after 48 hours [36].

This prostaglandin metabolite also has anti-inflammatory and anti-nociceptive effects that are TRPA1-independent. For example, Napimoga et al. (2008) reported the anti-nociceptive property of 15d-PGJ_2_ is mediated in part by PPARγ and peripheral opioid receptors, as antagonists targeting both receptors inhibited effects of 15d-PGJ_2_ against PGE_2_-induced “hypernociception.” This study also examined the anti-nociceptive effect of 15d-PGJ_2_ in the formalin model of temporomandibular joint inflammation and ipl. delivery of carrageenan, finding that it attenuated mechanical “hypernociception” in both models. They hypothesize that increases in the macrophage population in the periphery contributed to the enhancement the anti-nociceptive effects of 15d-PGJ_2_[19]. Whereas we are examining its action in strictly the pain neuraxis involving primary TRPA1-expressing peripheral nociceptors, perhaps the effects of 15d-PGJ_2_ on macrophages and/or opioids are downstream of signaling through primary afferents expressing TRPA1. For instance, activation of TRPA1-expressing nociceptors could result in their release of factors that contribute to an immune response (i.e. 15d-PGJ_2_ release) or endogenous opioid signaling.

### Differential behavioral and cellular effects of TRPA1 agonists

Previously published studies characterizing single-dose AITC-induced nocifensive responses in mice have utilized injections of 0.1-0.75% (10–75 mM, ipl.), while most behavioral desensitization studies using repeated AITC stimuli have utilized ≤ 20 mM. (e.g. [3,26,27]). Therefore, we compared behavioral desensitization to repeated AITC- and 15d-PGJ_2_-stimuli at two concentrations within this range. Using our dosing protocol, nocifensive responses to AITC did not desensitize. In contrast, 15d-PGJ_2_ evoked a significant desensitization to itself and AITC. Unlike previous studies using multi-dose protocols, we have administered agonists 1 h apart, which might account for the differences between our results and those of other studies, which report desensitization [26,29,37] or sensitization [27] by AITC. We hypothesize that the difference in the effects of 15d-PGJ_2_ and AITC on heterologous and homologous inhibition might result via differential acute and pharmacological desensitization of TRPA1 *in vivo* produced by the two agonists at various concentrations. Furthermore, topically applied and injected AITC causes an inflammatory response, including plasma extravasation and tissue edema, while there is no evidence supporting that 15d-PGJ_2_ promotes such responses [38,39].

To our knowledge, this is the first study to closely examine bidirectional cross-inhibition of DRG neurons by exposure to two different TRPA1 agonists. The mechanisms underlying the different effects of AITC and 15d-PGJ_2_ remain unclear (see also below). Since Ca^2+^ imaging does not directly measure desensitization, electrophysiological and further cellular studies are warranted to directly measure the mechanisms of desensitization, as well as desensitization kinetics, stimulus- and concentration-dependence. Key to our conclusions regarding inhibition of TRPA1 signaling by 15d-PGJ_2_, we have attempted to make our stimulus and recording approach(es) as systematic as possible in order to reveal differential modulation of TRPA1 by 15d-PGJ_2_ vs. AITC. First, our review of the available literature indicated to us that ~100 μM concentrations of 15d-PGJ_2_ and AITC are saturating in calcium imaging analyses of cultured sensory neurons [7,40]. We have confirmed these findings using our DRG culture and stimulus protocols. Similar to previously published EC_50_ data derived from heterologous expression studies of TRPA1 channels, we found 15d-PGJ_2_ and AITC to be of similar potency in their activation of TRPA1-expressing DRG neurons [6-8,11,40].

In DRG neurons, we observed strong homologous desensitization of AITC responses and cross-desensitization of 15d-PGJ_2_ in response to a pre-pulse of 100 μM AITC. Similarly, 100 μM 15d-PGJ_2_ inhibited subsequent responses to itself and to 100 μM AITC. Our results agree with those of Taylor-Clark et al. who reported that pre-treatment of trigeminal sensory neurons with 100 μM AITC significantly diminished subsequent responses to both 100 μM AITC and 15d-PGJ_2_[8]. While the majority of our studies examined the inhibitory efficacy of 100 μM 15d-PGJ_2_, we also investigated cross- and homologous desensitization using 50 μM concentrations of these compounds. We found that 50 μM AITC no longer exhibited these properties, whereas 15d-PGJ_2_ did at this lower concentration. Our data evince different magnitudes of homologous and heterologous inhibition evoked by these two compounds, each with steep time- and concentration-dependence, again the mechanisms of which remain uncertain.

A number of reports have demonstrated that similar to heat- and capsaicin-activated TRPV1, TRPA1 exhibits strong desensitization and tachyphylaxis [1], [29,40][41]. However, a more recent study partially refutes these data, showing that TRPA1 also undergoes sensitization and trafficking to the plasma membrane in response to AITC and inflammatory mediators *in vitro* and *in vivo*[27]. On balance, the results of our current study and those of previous studies reveal that sensitization/desensitization could be dependent on many variables, including agonist concentration, stimulus sequence, stimulus duration and inter-stimulus interval.

TRPV1 and TRPA1 are co-expressed in DRG neurons with TRPA1 labeling a subset of TRPV1 neurons [1]. Studies of the two channels in CHO cell overexpression and DRG neurons show that when co-expressed, TRPA1 undergoes heterologous and homologous desensitization via CAP (TRPV1 agonist) and AITC stimuli, respectively. A proposed mechanism of TRPA1 desensitization in this manner is through TRPV1-directed internalization [26,29]. It is intriguing that AITC is able to inhibit subsequent behavioral and neuronal responses to CAP, but 15d-PGJ2 (at the concentrations we have tested) is not, thus further highlighting the differential effects of these two TRPA1 agonists. Similar to Taylor-Clark et al. but in contrast to the findings of other groups, we find that pre-treatment with a TRPA1 agonist (in our case, 15d-PGJ_2_) in sensory neurons does not inhibit subsequent responses to CAP [8]. Correspondingly, we also did not observe behavioral desensitization to CAP as a result of 15d-PGJ_2_ pre-treatment, indicating that the inhibition is TRPA1 specific.

Although both compounds are electrophiles that activate TRPA1 via covalent modification of key cysteine (or other) residues, it is quite plausible that AITC and 15d-PGJ_2_ activate the channel differently, perhaps via differential modulation of required cysteines. For instance, there is indirect evidence that the cysteine required for 15d-PGJ_2_ activation in human TRPA1 is different than that required for activation by other compounds [11]. Perhaps also the two compounds induce differential trafficking to the plasma membrane [see [29] that is dependent on concentration. Finally, intracellular signaling cascades upon TRPA1 activation could be ligand-dependent, but this has never been shown. These possibilities raise an interesting avenue of research and could be addressed in mutagenesis studies combined with approaches such mass spectroscopy.

### TRPA1 and mechanosensitivity

We hypothesize that sufficient doses of 15d-PGJ_2_ effectively inhibit TRPA1 by desensitizing the channel. By applying this concept in our behavioral approaches to understanding the modulation of TRPA1 by 15d-PGJ_2_ in the CFA model of inflammatory hypersensitivity, we have essentially obtained the same results using a channel agonist as those studies which have utilized channel antagonists [21-23,42]. While 15d-PGJ_2_ had no effect on naïve mechanical thresholds nor perturbed the development of mechanical hypersensitivity, it inhibited established inflammatory mechanical hypersensitivity in WT, but not TRPA1^−/−^ mice. Although it appears that TRPA1 expression in peripheral mechanonociceptors is not required for the development of mechanical hypersensitivity, it is possible that sustained mechanical hypersensitivity results in part through an autocrine or paracrine positive feedback mechanism involving TRPA1. As TRPA1 is activated by products of cellular stress, factors released by mechanical damage could feed back on the channel. However, this possible signaling pathway does not resolve the conundrum of why TRPA1^−/−^ mice develop and maintain mechanical hypersensitivity, but TRPA1 antagonists can inhibit inflammatory and neuropathic hypersensitivity via “on-target” mechanisms.

### Conclusions and future directions

A goal of our studies was to determine whether 15d-PGJ_2_, or any strongly desensitizing TRPA1 agonist, could be useful as a pain therapeutic. For example, for a similar channel TRPV1, capsaicin is the active ingredient in over-the-counter and prescription topical treatments and provides effective relief of joint pain based on its ability to desensitize TRPV1-expressing fibers. Thus, TRPA1 agonists could serve a similar therapeutic role. Here we describe experiments in which we administered 15d-PGJ_2_, beginning 1 day after the induction of inflammation. Our dosing strategy repeatedly produced a ~2 h block of mechanical hypersensitivity as shown using the von Frey test. Our studies show the effectiveness of administering such a dose after, but not before, the development of inflammation or neuropathic injury. This is in accordance with the nature of pain treatment, which is given *after* a patient reports discomfort from inflammation or injury. With the route and concentration we tested in our multi-dose experiment (15 mM, ipl.), 15d-PGJ_2_ initially causes nocifensive responses, but appears to have no pro-inflammatory or neurotoxic effects on TRPA1-expressing neurons. Future studies will further test the efficacy of 15d-PGJ_2_ as a therapeutic by determining a dose or route of administration (e.g. oral or topical application) that causes minimal pain, while providing analgesic benefits.

## Methods

### Animals

Experiments were performed in accordance with the policies and recommendations of the National Institutes of Health and the International Association for the Study of Pain. Protocols were approved by the Animal Care and Use Committee of Washington University School of Medicine (St. Louis, MO). Mice were housed at ambient (22-25°C) temperature with a 12 h light–dark cycle. Cellular and behavioral assays were performed using 8- to 12-wk-old male C57BL/6 J and TRPA1 knockout mice. The TRPA1^−/−^ strain was originally obtained from Kelvin Y. Kwan and David P. Corey [3]. Heterozygous F2 crosses were set and behavioral tests carried out on male WT and TRPA1^−/−^ littermates of 8–12 weeks of age.

### Behavioral assays

Animals were placed in individual Plexiglas boxes (15X11X11 cm) either on a flat surface covered with an absorbent liner (chemical agonist tests) or on a wire mesh platform (von Frey tests) and acclimated for 1–2 h to the testing environment—an individually lighted, temperature-controlled room (22-25°C) equipped with a white noise generator. Experimenters were blind with respect to treatment and genotype. Sample sizes are indicated in Figure legends.

#### Compound tests

For dose–response experiments, mice were injected on the plantar surface of the right hindpaw with 10 μL of 0.15 (.474 μg), 1.5 (4.74 μg) or 15 mM (47.4 μg) 15d-PGJ_2_ diluted in 5% EtOH, 0.9% NaCl (vehicle). Nocifensive behavior (licking and deliberate lifting and shaking of the injected hindpaw) was recorded at 5- to 10-min intervals for 1 h. For multi-dose 15d-PGJ_2_ experiments, mice received two ipl. injections of 10 μL 1.5 or 15 mM 15d-PGJ_2_ 1 h apart. For multi-dose AITC experiments, mice received two ipl. injections of 10 μL of either 0.15 mM (0.0015%), 1.5 mM (0.015%) or 15 mM, (0.15%) AITC (diluted in mineral oil) 1 h apart. Nocifensive behavior was recorded at 5- to 10-min intervals for 1 h.

To test for 15d-PGJ_2_-induced inhibition of nociceptive responses to AITC or CAP, groups of mice were first injected on the plantar surface of the right hindpaw with 10 μL of 1.5 or 15 mM 15d-PGJ_2_ or vehicle. 1 h later, an injection of 10 μL AITC or 10 μL CAP (1.5 μg) was administered to the same paw. For these tests, nocifensive behaviors (licking and deliberate lifting and shaking of the injected hindpaw) in response to the second injection were recorded at 5- to 10-min intervals until they completely subsided—1 h for AITC and 15 min for CAP. We also tested whether ipl. injection of 10 μL 15 mM AITC was able to inhibit subsequent responses to an injection of 15 mM 15d-PGJ_2_ administered to the same paw 1 h later.

To determine whether inhibition of AITC responses by 15d-PGJ_2_ were locally mediated, we also performed a set of experiments in which we injected the left hindpaw of two groups of mice with 15 mM 15d-PGJ_2_ or vehicle and the right hindpaw with 15 mM AITC 1 h later. Nocifensive behaviors in response to the second AITC injection in the right hindpaw were compared between the two groups injected with 15d-PGJ_2_ or vehicle in the left hindpaw.

#### Mechanical hypersensitivity tests

We measured mechanical sensitivities with calibrated von Frey hairs using the up-and-down method modified for mice [43]. We first investigated whether an ipl. injection of 10 μL 1.5 or 15 mM 15d-PGJ_2_ vs. vehicle had any effect on basal mechanical sensitivity. These experiments were performed by acquiring baseline von Frey measurements and then injecting 15d-PGJ_2_ or vehicle (5% EtOH, 0.9% NaCl) into the right hindpaw. Subsequent von Frey measurements of the ipsi- and contralateral hindpaws were taken at 1, 2, 6, 24 and 48 h post-injection.

In a separate set of experiments, we evaluated the effects of 15d-PGJ_2_ on CFA-induced inflammatory mechanical hypersensitivity. On day 0, baseline sensitivity to von Frey stimulation was measured prior to ipl. injection of 10 μL CFA to the right hindpaw of mice. 24 h after CFA injection (day 1), von Frey sensitivities were measured again to determine the level of mechanical hypersensitivity in the CFA vs. vehicle groups. At the conclusion of these von Frey measurements, CFA-injected mice were randomly assigned to two groups and received either an ipl. injection of 10 μL 15 mM 15d-PGJ_2_ or vehicle to the right hindpaw. One h after this second injection, von Frey measurements were taken again at 1, 2, 4, 6 and 24 h post-injection. To examine the effect of repeated dosing of 15d-PGJ_2_, we performed this procedure as above through day 1 and then repeated it on days 2–4. Therefore, in this set of experiments, we made von Frey measurements on days 0–4 at the following time points: baseline (day 0), 24 h post-CFA (or vehicle) injection (day 1) and then 1, 2 and 24 h post-15d-PGJ_2_ (or vehicle) injection (days 1–4). To test for maintenance of nociceptive responses to TRPA1 activation, all mice were administered an ipl. injection of 10 μL 50 mM AITC at the conclusion of our repeated 15d-PGJ_2_ dosing experiment.

In another set of experiments on groups of WT and TRPA1^−/−^ mice, we repeated this procedure as in day 1 above, but only up to 2 h post-15d-PGJ_2_ or vehicle injection (observed time of maximal 15d-PGJ_2_ effect on CFA-induced mechanical hypersensitivity).

#### Heat hypersensitivity test

Thermal sensitivity was measured using the Hargreaves test [44]. Mice were placed in Plexiglas chambers on a heated (30°C) glass surface and acclimated to the apparatus and testing environment for 1 h. Radiant heat of constant intensity was applied to the plantar surface of the ipsilateral hindpaw using a 390 G Plantar Test Apparatus (IITC Life Sciences, Woodland Hills, CA). Withdrawal latency was calculated as the time from stimulus initiation until paw withdrawal with a 20 s cutoff to prevent tissue damage. The test was repeated 3 times (~15 min between tests of same paw) and the withdrawal latencies of the 3 repetitions averaged.

### DRG culture and calcium imaging

Dissociated DRG cultures derived from male C57BL/6 J mice were prepared and plated onto polyornithine- and laminin-coated coverslips as described [45,46]. For each experimental condition we performed imaging on at least 2 separate cultures derived 4 animals. These cultures yielded ~ 8 coverslips each on which we performed paired comparisons (e.g. 100 μM AITC vs. 100 μM 15d-PGJ2). Agonist-induced responses of DRG neurons were analyzed using Fura-2 (Invitrogen) ratiometric calcium imaging 24 h after plating. Wash, vehicle and compound solutions contained HEPES buffered saline with 2 mM calcium. We exposed cells to 50 mM potassium at the end of experiments as a control for viable neurons. We defined neuronal responses as ≥ 20% increase of the Fura-2 ratio from baseline, with the exception of some dose-inhibition experiments (see Results). For all experiments, the compound exposure and wash-out durations are noted in each Figure and/or corresponding legend.

The concentrations of compounds used in cross-desensitization studies were as follows: 50 μM and 100 μM 15d-PGJ_2_, 50 μM and 100 μM AITC and 1 μM CAP. We compared the responses of neurons to AITC and CAP from the 15d-PGJ_2_-treated groups to those of the vehicle-treated (0.1% DMSO) groups. To investigate the duration of desensitization of the AITC response induced by pre-exposure to 15d-PGJ_2_, we exposed the cells to either 50 μM AITC or 100 μM 15d-PGJ_2_ for 1 min, washed continuously with buffer solution for up to 16 min and then re-exposed the cells to 50 μM AITC.

Finally, to test whether inhibition of AITC responses by 15d-PGJ_2_ was mediated specifically by TRPA1, we utilized the TRPA1-specific antagonist HC-030031. We compared the magnitude and frequency of AITC responses after 15d-PGJ_2_ exposure vs. 15d-PGJ_2_ exposure in the presence of the antagonist.

### Statistical analysis

Student’s t-tests or Mann Whitney U-tests were used to analyze data where appropriate; the tests used are indicated in the Figure legends. Repeated measures analysis of variance (RMANOVA) with Bonferroni post-hoc correction was used to test changes in nociceptive behaviors over time in the AITC and CAP inhibition tests and in the CFA model of inflammatory hypersensitivity. All behavioral data were analyzed using Prism 5.0. Data from DRG studies were analyzed using OriginPro 8.1 (OriginLab Corp., Northampton, MA 01060) and Prism 5.0 (GraphPad Software Inc., San Diego, CA). In all studies, statistical significance is set at *p* < 0.05 and data are presented as mean ± S.E.M.

### Compounds

AITC, CAP and CFA were purchased from Sigma Chemical Company. 15d-PGJ_2_ was purchased from Enzo Life Sciences. HC-030031 was purchased from Santa Cruz. For behavioral experiments, stock solutions of 15d-PGJ_2_ or CAP were dissolved in ethanol and diluted in normal saline solution (0.9% NaCl) so that the final concentration of EtOH was ≤ 5%. Solutions of AITC were prepared by suspending directly in mineral oil. For calcium imaging experiments, stock solutions of 15d-PGJ_2_, AITC and CAP were dissolved in DMSO or ethanol and used at a final solvent concentration of ≤ 0.1% in buffer solution.

## Abbreviations

TRPA1: Transient receptor potential cation channel subfamily A member 1; 15d-PGJ_2_: 15-Deoxy-Δ12, 14-prostaglandin J_2_; AITC: Allyl isothiocyanate; CAP: Capsaicin; WT: Wildtype; TRPA1^−/−^: TRPA1 knockout (null).

## Competing interests

The authors have no financial or non-financial competing interests.

## Authors’ contributions

YW performed behavior experiments and statistical analyses of these. PAB assisted with the design and execution of behavioral studies with YW as well as performed Ca^2+^ imaging studies. MEB performed and analyzed AITC and 15d-PGJ_2_ desensitization studies in calcium imaging experiments. TBD and MJS performed and analyzed Ca^2+^ imaging studies of AITC and 15d-PGJ_2_ dose–response, cross-desensitization and antagonist studies. EQH, AMF, MW, VMW prepared all DRG cultures utilized by the GMS lab, performed all mouse husbandry and PCR genotyping of TRPA1^−/−^ line and assisted with behavioral experiments. EAK performed and analyzed 15d-PGJ2-induced desensitization of CAP calcium imaging studies. CLS provided technical supervision of Ca^2+^ imaging studies, assisted in the design and analysis of these and helped to draft the manuscript. GMS conceived of the study, performed and supervised Ca^2+^ imaging and behavioral experiments, produced Figures and wrote the manuscript. All authors read and approved the final manuscript.
